# Effects of tryptamine on duckweed growth

**DOI:** 10.3389/fpls.2025.1625939

**Published:** 2025-07-08

**Authors:** Qiqi Di, Wenqian Han, Yujie Han, Sizheng Liu, Yi Hu, Ziyang Qu, Yumeng Jiang, Weibo Sun, Ting Qiu, Lin Yang

**Affiliations:** ^1^ Tianjin Key Laboratory of Animal and Plant Resistance, College of Life Sciences, Tianjin Normal University, Tianjin, China; ^2^ Tsinghua-Peking Center for Life Sciences, College of Life Sciences, Tsinghua University, Beijing, China

**Keywords:** tryptamine, *Lemna turionifera* 5511, growth regulation, photosynthesis, oxidative stress, hormonal balance

## Abstract

**Introduction:**

Plant growth regulation involves complex biochemical and signaling pathways. Tryptamine (Try), a polyamine derived from tryptophan, has been implicated in plant growth and stress responses, yet its specific regulatory mechanisms have not been fully understood.

**Methods:**

This study investigates the physiological and molecular effects of Try on Lemna turionifera 5511, focusing on its role in growth regulation, photosynthesis, and hormonal balance. Our findings reveal that Try content increases in overgrown duckweed, suggesting its involvement in aging and stress responses. Exogenous Try application at concentrations ranging from 50 to 200 μM resulted in dose-dependent growth inhibition, with 150 μM Try significantly reducing growth rate, leaf area, and chlorophyll content.

**Results:**

The Chlorophyll a (Chla) and Chlorophyll b (Chlb) levels were decreased by 37.5% and 40.43%, respectively. Try treatment also negatively impacted photosynthesis, as evidenced by reduced chlorophyll fluorescence parameters and downregulation of 16 photosynthesis-related genes. Additionally, Try induced oxidative stress, increasing reactive oxygen species (ROS) and peroxidase (POD) activity by 9.17% and 10.11%, respectively. While modulating endogenous hormone levels, particularly increasing abscisic acid (ABA) and decreasing cytokinin (CTK) content by 23.58% and 17.55%. Moreover, transcriptomic analysis revealed an upregulation of auxin (IAA) metabolism-related enzymes by Try addition. Meanwhile, changes in the expression of genes related to the tryptophan metabolism pathways indicate a metabolic change associated with aging.

**Discussion:**

These results highlight the complex role of Try in regulating duckweed growth and stress responses, suggesting its potential as a regulatory molecule in plant development. Further research is needed to elucidate the molecular mechanisms underlying the influence of Try and its applications in agriculture and environmental management.

## 1 Introduction

Plants synthetize an extensive array of low molecular weight organic compounds, broadly classified as primary metabolites, secondary metabolites, or hormones (Erb and Kliebenstein, 2020), which play diverse roles in plant growth, development, and stress responses. Plant growth itself is a highly complex biological process governed by intricate networks of biochemical and signaling pathways (Olas et al., 2020). As plants reach certain developmental stages, their growth rates typically decrease, yet the inside regulatory mechanisms underlying this phenomenon, including the production and activity of these compounds that are tightly regulated through complex metabolic networks, remain elusive. Polyamines, a class of ubiquitous small aliphatic polycations in eukaryotic organisms (Seifi and Shelp, 2019), are characterized by their polymer-like carbon chain structures containing multiple amino groups (Ćavar Zeljković et al., 2024). Polyamines function as secondary metabolites in plants (Ćavar Zeljković et al., 2024) and play significant roles in various physiological processes, including seed germination, tuber dormancy break, root development, floral initiation and development, senescence delay, organogenesis, and stress responses (Yousefi et al., 2019). Among these polyamines, Try represents a particularly interesting yet understudied compound whose role in plant growth regulation remains poorly understood.

Tryptamines, a class of compounds derived from the essential amino acid tryptophan, are characterized by an indole ring structure consisting of a benzene ring fused to a pyrrole ring, with an amino group attached to a 2-carbon side chain (Zohairi et al., 2023). Try biosynthesis occurs through tryptophan metabolism via the Try biosynthetic pathway. These tryptophan-derived monoamines share structural similarities with 5-hydroxytryptamine (5-HT) (Bhattarai et al., 2018), and serve as crucial biosynthetic precursors for numerous natural alkaloids. Furthermore, tryptamines are frequently employed as fundamental chemical building blocks in the total synthesis of biologically active compounds and pharmaceutically important substances (Simonetti et al., 2021). The enzymatic conversion of Try to 5-hydroxytryptamine, mediated by tryptamine 5-hydroxylase (T5H), represents a critical biochemical pathway. 5-HT, a well-established neuromodulator, plays a pivotal role in the mechanism of various psychoactive drugs. Its complex interactions with other neuromodulators, including norepinephrine, dopamine, and oxytocin, significantly influence human subjective experiences and mental health (Ligneul and Mainen, 2023). Beyond its neurological significance, Try also serves as a key metabolite in tryptophan catabolism. Try plays an indispensable role in numerous physiological processes, particularly in IAA synthesize. However, the dynamics of Try levels during plant growth remain poorly characterized.

Auxin (IAA), a morphogen-like substance, is essential for plant growth and development, regulating processes such as branching, gravitropism, phototropism, and seed development (Benková et al., 2003; Perico et al., 2022). In higher plants, tryptophan-dependent IAA biosynthesis primarily proceeds through four well-characterized pathways: the indole-3-pyruvate (IPyA) pathway (Stepanova et al., 2008), the tryptamine (YUCCA) pathway (Zhao et al., 2001), the indole-3-acetaldoxime (IAOx) pathway (Bartel et al., 2001), and the indole acetamide pathway (Pollmann et al., 2002). While Try plays a crucial role in IAA synthesis under normal physiological conditions, both relevant literature (Wan et al., 2018) and our previous research revealed a paradoxical relationship between Try concentration and plant growth, with 150 μM of exogenous Try identified as the optimal concentration for inhibiting duckweed growth. This finding aligns with studies suggesting that elevated IAA levels may initiate senescence processes (Casanova-Sáez et al., 2021). Try, as one of these plant metabolites, has not been thoroughly investigated in terms of its endogenous levels and physiological effects. Our study focus on differential Try accumulation patterns between senescent and young duckweed, aiming to reveal that the change in Try content may affect the growth of plants.

Duckweeds, described as the “simplest and smallest of flowering plants” (Hillman, 1961), have small stature and morphological simplicity. As one of the fastest-growing higher plants, duckweeds can quickly cover water surfaces (Ziegler et al., 2015). This rapid growth is beneficial for fundamental research as it enables the quick accumulation of large sample sizes. Studies have shown that, depending on the species and culture medium, the population size of duckweed doubles within 1.43 to 4.54 days, thus achieving high biomass yield in a short period of time (Kang et al., 2025). The growth characteristics of duckweed make it an ideal model for studying the regulation of plant growth. Existing studies have shown that the exogenous addition of secondary metabolites such as ABA can have an impact on the growth of duckweed (Utami et al., 2018). However, the relationship between the growth of duckweed and the exogenous application of Try remains unclear. In this study, we employ *Lemna turionifera* 5511 as a model system to investigate the physiological role of Try. Our research aims to address three specific objectives: (i) to examine the relationship between endogenous Try levels and the reduced growth rate of duckweed; (ii) to evaluate the effects of exogenous Try application on duckweed growth; and (iii) to analyze the impact of exogenous Try on the IAA metabolic pathway. Through these investigations, we aim to elucidate the complex interplay between Try metabolism and plant growth regulation.

## 2 Materials and methods

### 2.1 Duckweed culture and try treatment settings

The duckweed used in this study (*Lemna turionifera* 5511) was originally obtained from the Fengchan river in Tianjin, China. The duckweed was cultivated according to our previous study (Wang et al., 2023). The culture condition was a temperature of 24°C/22°C at day/night, under 16 h light/8 h dark cycle, and with a light intensity of 95 µmol m^-2^·s^-1^. The duckweed was treated with 50, 100, 150 and 200 μM Try for 9 days. The treatment with or without 150 μM Try was used for the following experiments.

### 2.2 Enzyme linked immunosorbent assay

The determination of peroxidase (POD), reactive oxygen species (ROS), abscisic acid (ABA), auxin (IAA), cytokinin (CTK) and tryptamine (Try) activities was performed using double-antibody sandwich enzyme-linked immunosorbent assay (ELISA) kits (Enzyme-linked Biotechnology Co., Shanghai, China). Plant samples from CK and Try groups were ground with 0.1 g of fresh weight in liquid nitrogen and then centrifuged with 1 mL of PBS to create a suspension. The supernatant was diluted 5 times before being used for sampling. By coating microplates with specific antibodies, followed by diluting supernatants, binding with HRP-conjugated secondary antibodies, developing the color reaction with TMB, and finally quantifying absorbance at 450 nm for results. The standard curve is:

POD: y=0.024x-0.0511   ROS: y=0.0125x+0.0835

ABA: y=0.0676x+0.1412   IAA: y=0.0742x+0.1168

CTK: y=0.0635x+0.0757   Try: y=0.343x+0.7281

### 2.3 Chlorophyll fluorescence parameter monitoring and photosynthetic pigment content determination

The Dual-PAM100 fluorometer from Waltz, Germany, was used to determine the duckweed chlorophyll fluorescence parameters including photosystem II complex (PSII), a photosynthetic unit in the thylakoid membrane, maximum quantum yield of PSII (Fv/Fm), quantum yield of PSII (Y (II)) and nonphotochemical quenching coefficient (qN). The samples were given a 30-min dark treatment prior to the measuring in order to reduce the amount of organic materials in the plant tissue before detection.

A total of 0.1 g of fresh duckweed from CK group and Try group were immersed in 10 mL of 95% alcohol within dark for 24 hours. The absorbance of the extract was measured at wavelengths of 645 nm and 663 nm using a multimode micro-plate reader (TECAN, Spark, Switzerland). Chlorophyll a (Chla), chlorophyll b (Chlb), and total chlorophyll (Chla+b) were calculated as follows:

Chla (mg/g)={(12.7A663-2.69A645)×10mL}/0.1g

Chlb (mg/g)={(22.9A645-4.68A663)×10mL}/0.1g

Chl(a+b) (mg/g)={(8.02A663 + 20.21A645)×10mL}/0.1g

### 2.4 RNA sequencing and analysis

Following 48 h of Try treatment, the RNA from both the CK and Try groups underwent sequencing and analysis at Novogene in Chaoyang, Beijing, China. Total RNA was collected using the QIAGEN Total RNA prep Pure Plant Kit (Beijing, China). The quality of the RNA was assessed using an Agilent 2100 Bioanalyzer (Santa Clara, CA, USA). Subsequently, the library was constructed and examined using the Illumina NovaSeq 6000 sequencing system (San Diego, CA, USA). Firstly, the integrity of the RNA extracted from the duckweed with two group was analyzed by agarose gel electrophoresis. A NanoPhotometer spectrophotometer was used to detect the purity of RNA. Secondly, total RNA (≥0.25 g) was used to prepare the library, and the effective concentration (≥2 nM) of the library was accurately quantified by qRT-PCR to ensure the quality of the library. Thirdly, clustering and sequencing index-coded samples clustering has been performed according to the manufacturer’s instructions. The library preparations were sequenced on an Illumina HiSeq platform (performed at Novogene Bioinformatics Technology Co., Ltd. Beijing, China). The gene functions were annotated according to Gene Ontology and KEGG databases. The DESeq R package (1.10.1) was used to analyze differential gene expression of “CK” and “Try_150 μM”. The criterion was that genes with adjusted p-value < 0.05 were designated as differently expressed. GO seq and KOBAS software were used to perform Gene Ontology functional enrichment and KEGG pathway enrichment analyzed on differential gene sets.

### 2.5 Statistical analysis

The data were presented as the mean ± SD triplicate. The area of frond was measured by imageJ. The graphs were prepared in Illustrator 2022 and Photoshop 2023. Microsoft Excel 2021 was used for data processing. GraphPad Prism 9 was used for Figure drawing. Statistical analyses were performed using Statistical Program for Social Science (SPSS). Statistical significance was assessed by independent samples t-test, with significant differences denoted by asterisks. (*: p < 0.05, **: p < 0.01, ***: p < 0.001, ****: p < 0.0001, ns: not significant).

## 3 Results

### 3.1 Influence of external application of Try on the growth of duckweed

**Figure 1:**
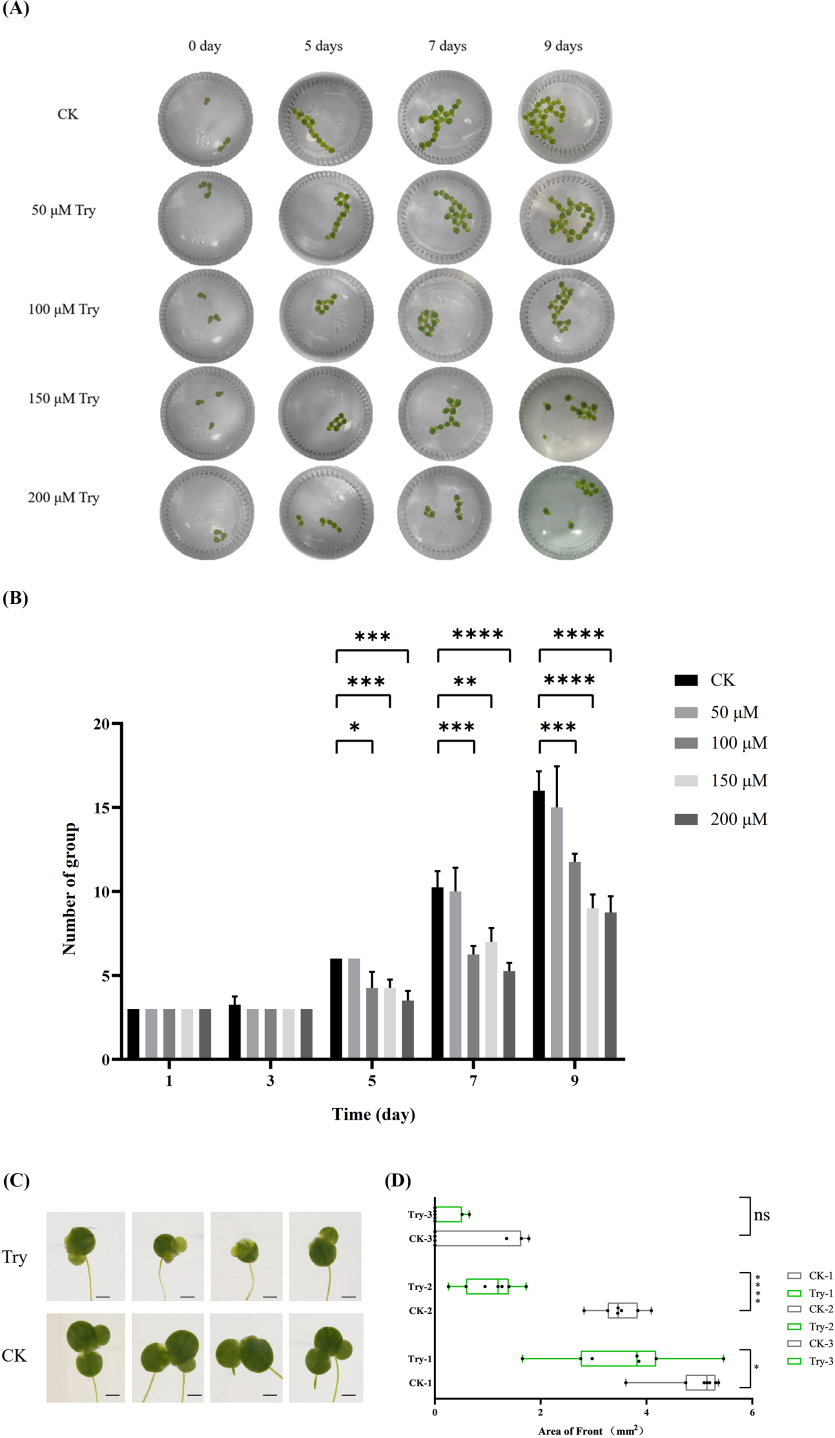
Influence of external application of Try on the growth of duckweed. **(A)** Leaf diagram; **(B)** Number of group; **(C)** Leaf morphology; **(D)** Leaf area. CK-1, Area of the largest leaf in the control group; CK-2, Area of the second largest leaf of the control group; CK-3, Area of the third largest leaf of the control group; Try-1, Area of the largest leaf in the Try treatment group; Try-2, Area of the second largest leaf of the control group; Try-3, Area of the third largest leaf of the control group. (*p < 0.05, **p < 0.01, ***p < 0.001, ****p < 0.0001, ns, not significant).

In order to study the effect of external application of Try on duckweed growth, we treated duckweed with different concentrations of Try, calculated its growth status, and then selected an optimal concentration for subsequent experiments. The results showed that the growth rate of duckweed decreased with the increase of exogenous Try (**Figure 1A**). On the fifth day of Try treatment, the 100 μM, 150 μM and 200 μM Try treatment groups showed significant changes compared with the blank control (CK) group (*p < 0.05*). The average growth rate of CK group was 6 plants per bottle, that of 50 μM group was 6 plants per bottle, that of 100 μM group was 4.25 plants per bottle, that of 150 μM group was 4.25 plants per bottle and that of 200 μM group was 3.5 plants per bottle. On the ninth day, this significant difference was even more pronounced (*p < 0.001*). At this time, the average growth of CK group was 16 plants per group, was 15 in 50 μM group (6.25% less than control), was 11.75 in 100 μM group (26.56% less than control), was 9 in 150 μM group (43.75% less than control) and was 8.75 in 200 μM group (45,31% less than control) (**Figure 1B**). The concentrations of 50 μM, 100 μM, 150 μM, and 200 μM Try were applied to the duckweed. We found that under 50 μM, 100 μM, the growth of duckweed showed no significant change compared to the CK group. And 150 μM Try addition lead to a decreased growth rate of duckweed. The treatment with or without 150 μM Try was used for the following experiments.

After treating duckweed with 150 μM Try for nine days, we observed the growth of duckweed leaves using a stereomicroscope. The pictures showed that the leaf edges of duckweed treated with Try showed yellowing and browning, and the hyalinization was more serious (**Figure 1C**). Based on this, we further measured the leaf area of duckweed in CK group and Try treated group. The leaf area of the first leaf in the Try treatment group decreased by 28.25% compared with the CK group, and the leaf area of the second leaf in the Try treatment group decreased by 69.91% compared with the CK group. The area of the first leaf and the second leaf in Try treatment were reduced significantly (*p < 0.05, p < 0.0001*) (**Figure 1D**). These results indicated that the phenotype of duckweed changed after external application of Try, indicating that Try could regulate the growth process of duckweed.

### 3.2 Influence of 150 μM concentration of Try on photosynthesis of duckweed

**Figure 2:**
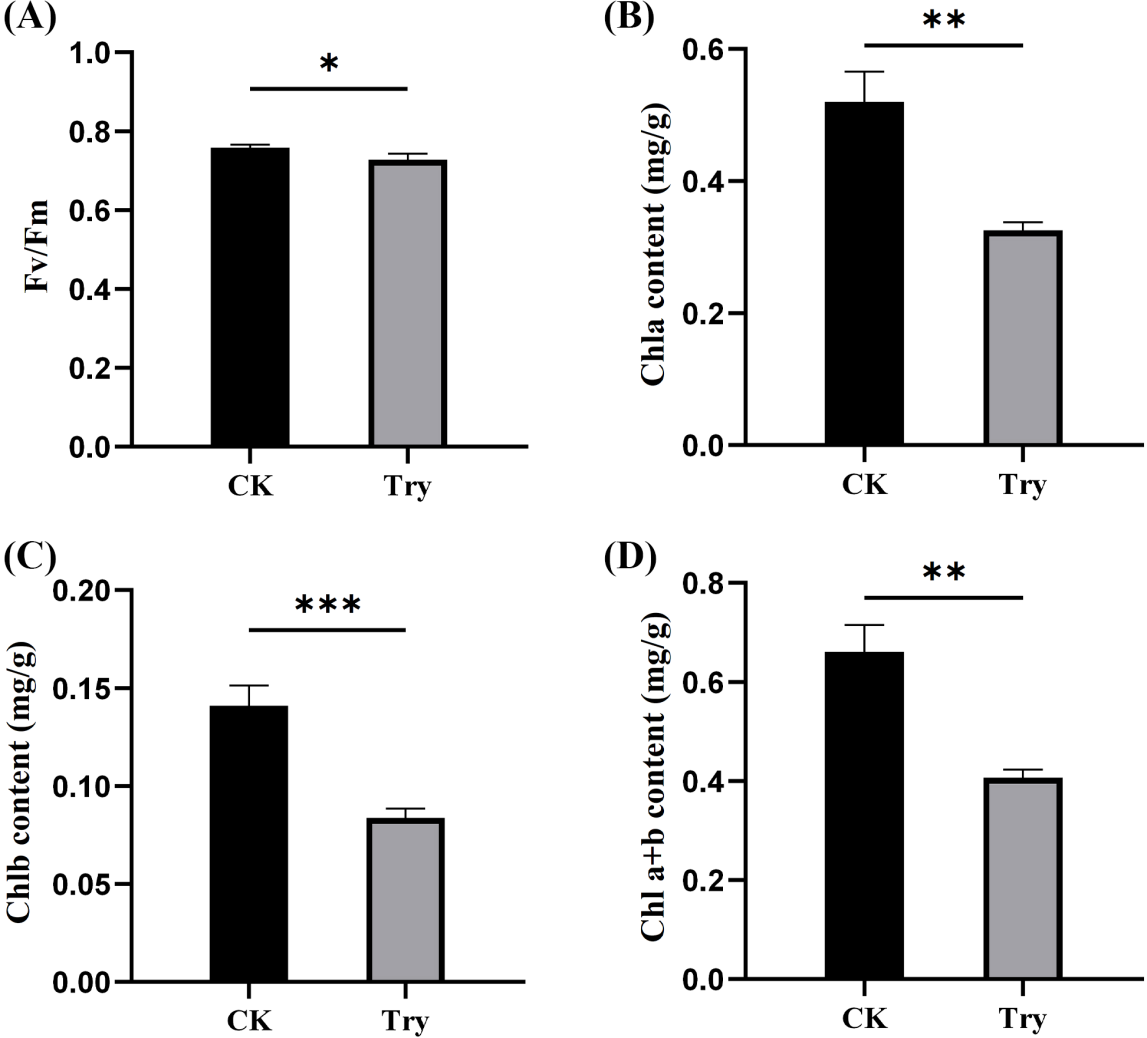
Influence of 150 μM concentration of Try on photosynthesis of duckweed. **(A)** maximum photochemical quantum efficiency of PSII (Fv/Fm); **(B)** chlorophyll a content; **(C)** chlorophyll b content; **(D)** total chlorophyll content. (*p < 0.05, **p < 0.01, ***p < 0.001).

To investigate the changes in photosynthesis of duckweed after external treatment with Try, Fv/Fm value and chlorophyll content of duckweed leaves were determined. We also measured at the genetic level to reveal the effect of Try on photosynthesis of duckweed. The results indicated that the 150 μM Try treatment significantly reduced the value of Fv/Fm by 3.96%, compared to the CK (*p < 0.05;* **Figure 2A**).

After processing for 8 days, the chlorophyll contents of CK and Try samples were determined. It was found that compared with CK group, the content of chlorophyll a, chlorophyll b and total chlorophyll in Try treatment group reduced by 37.50%, 40.43% and 37.97%, respectively (*p < 0.01;* **Figures 2B–D**).

Moreover, the changes of gene expression levels showed that the Try treatment affected protein content by influencing gene expression levels. The levels of gene expression almost all have reduced (**Table 1**). These results indicated that exogenous Try could regulate the growth of duckweed by affecting photosynthesis.

**Table 1:** Changes in gene expression levels related to photosynthesis and antenna proteins.

Description	Gene ID	Tryptamine Readcount	CK readcount	log_2_ fold change	pvalue	padj
light-harvesting complex II chlorophyll a/b binding protein 7	Cluster-3253.9432	418.09	1125.84	-1.43	3.5759E-68	1.4369E-66
light-harvesting complex I chlorophyll a/b binding protein 5	Cluster-3253.14792	396.37	2795.45	-2.82	1.7533E-291	7.1029E-289
ferredoxin–NADP^+^ reductase	Cluster-3253.10798	1627.66	4821.59	-1.57	4.2577E-54	1.3282E-52
cytochrome b6-f complex iron-sulfur subunit	Cluster-3253.11313	17529.56	48503.19	-1.47	1.1969E-55	3.8602E-54
photosystem II oxygen-evolving enhancer protein 2	Cluster-3253.12733	343.90	1109.96	-1.69	2.02E-67	8.01E-66
photosystem II oxygen-evolving enhancer protein 3	Cluster-3253.8578	1047.26	3005.57	-1.52	1.42E-75	6.31E-74
photosystem II oxygen-evolving enhancer protein 3	Cluster-3253.15637	1791.77	3789.21	-1.08	1.94E-54	6.09E-53
photosystem II 22kDa protein	Cluster-3253.11131	6218.14	44708.50	-2.85	0	0
photosystem II 13kDa protein	Cluster-3253.8696	1497.77	4939.03	-1.72	5.47E-108	3.81E-106
photosystem I subunit VI	Cluster-3253.16640	91.60	191.83	-1.06	2.86E-11	2.26E-10
Plastocyanin	Cluster-3253.14410	79.74	4043.95	-5.66	0	0
Plastocyanin	Cluster-3253.11589	23940.01	65090.69	-1.44	1.81E-50	5.22E-49
Ferredoxin	Cluster-3253.10665	21886.30	87470.89	-2.00	1.91E-156	2.30E-154
F-type H^+^/Na^+^-transporting ATPase subunit beta	Cluster-3253.13551	28.23	62.76	-1.14	4.60E-05	0.00021621
F-type H^+^/Na^+^-transporting ATPase subunit alpha	Cluster-3253.5449	113.83	245.31	-1.11	4.21E-17	4.55E-16
F-type H^+^-transporting ATPase subunit delta	Cluster-3253.12398	11662.77	23387.87	-1.00	7.61E-43	1.83E-41

### 3.3 Influence of endogenous Try content on Try metabolism and growth state of duckweed

**Figure 3:**
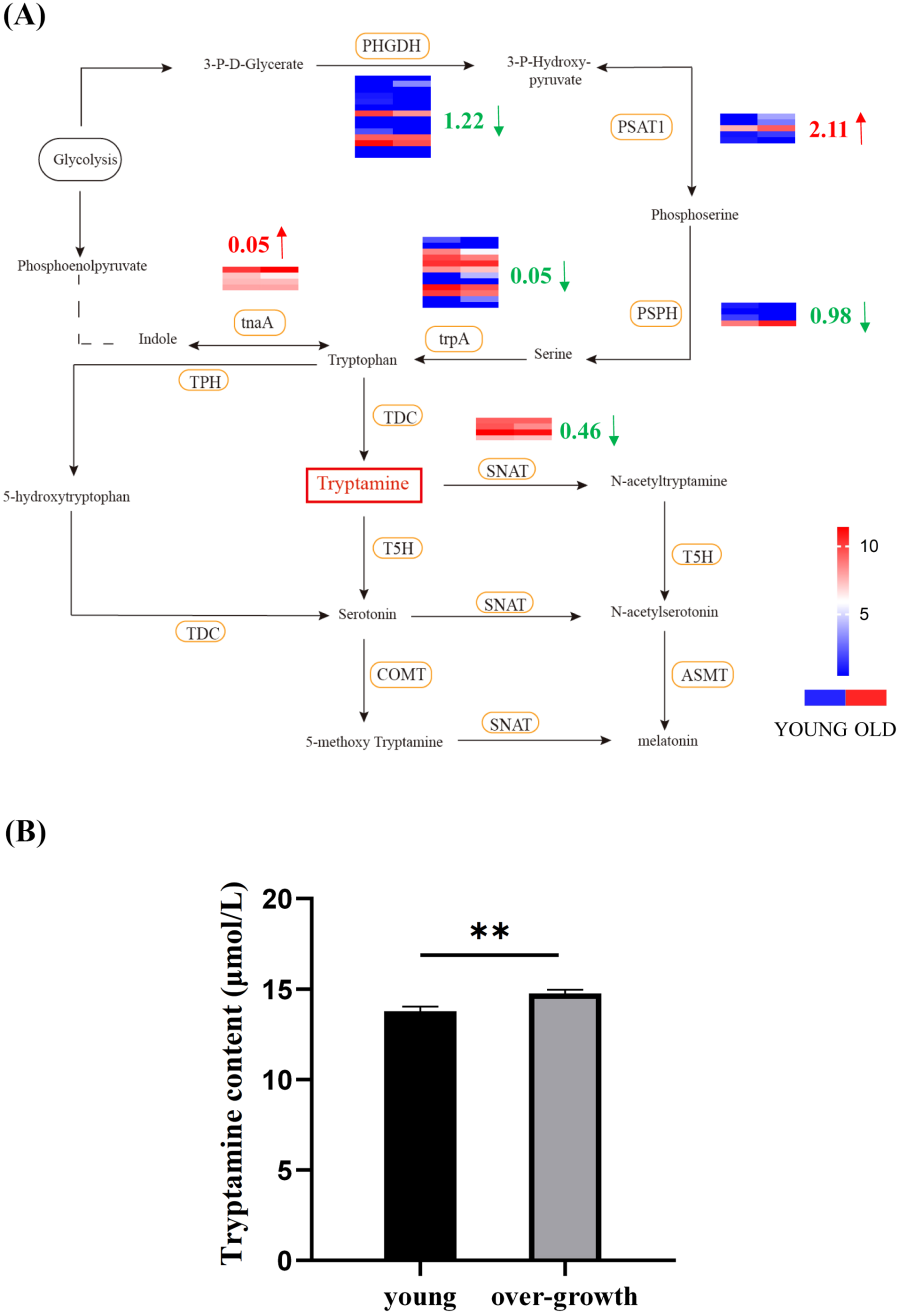
Influence of endogenous Try content on Try metabolism and growth state of duckweed. **(A)** Heatmap correlation between the Try relation metabolic pathway in young and senescent duckweed. Red arrows represent up-regulation, green arrows represent down-regulation. The legend colors in this figure were changed from red to blue, reflecting a decrease in log_2_ fold change values (red: high expression; blue: low expression). The column on the left of the heat map represents young duckweed and the column on the right represents senescent duckweed. The heatmap data is the log_2_ value of the readcount. PHGDH, phosphoglycerate dehydrogenase; trpA, tryptophan synthase alpha chain; PSAT1, Phosphoserine Aminotransferase 1; PSPH, phosphoserine phosphatase; TDC, tryptophan decarboxylase; tnaA, tryptophanase; SNAT, arylalkylamine N-acetyltransferase; COMT, Catechol-O-MethylTransferase; TPH, tryptophan 5-monooxygenase; T5H, tryptamine 5-hydroxylase; ASMT, acetylserotonin O-methyltransferase; SNAT, Serotonin N-Acetyltransferase; TPH, Tryptophan Hydroxylase; **(B)** The content of Try in senescent and young duckweed, asterisks indicate significant differences between senescent and young samples. (**p < 0.01).

To elucidate the role of Try in the development process of duckweed, we performed RNA-seq analysis to investigate the differential expression of genes involved in Try metabolism between young and senescent duckweed (**Figure 3A**). It was found that the three enzymes directly related to Try metabolism were: TDC, T5H, and SNAT. In senescent duckweed, the expressions of TDC and T5H did not change significantly, while the expression of SNAT was significantly downregulated. The expression of SNAT was downregulated by 0.46 log_2_ Fold Change. However, in the metabolic pathways related to Try, the expression of other enzymes had undergone significant changes. The expression of PHGDH was downregulated by 1.22 log_2_ Fold Change, that of PSPH was downregulated by 0.98 log_2_ Fold Change and that of trpA was downregulated by 0.05 log_2_ Fold Change. The expression of tnaA was upregulated by 0.05 log_2_ Fold Change, and the expression of PSAT1 was upregulated by 2.11 log_2_ Fold Change. In order to understand more clearly the content of Try in young and senescent duckweed, we examined the Try content in young and senescent duckweed respectively (**Figure 3B**). The Try content in the senescent duckweed was 14.76 µmol/L, nearly 7% higher than that in the control group (13.78 µmol/L). These results indicated that Try accumulates in senescent duckweed.

### 3.4 Influence of 150 μM Try on endogenous hormones of duckweed

**Figure 4:**
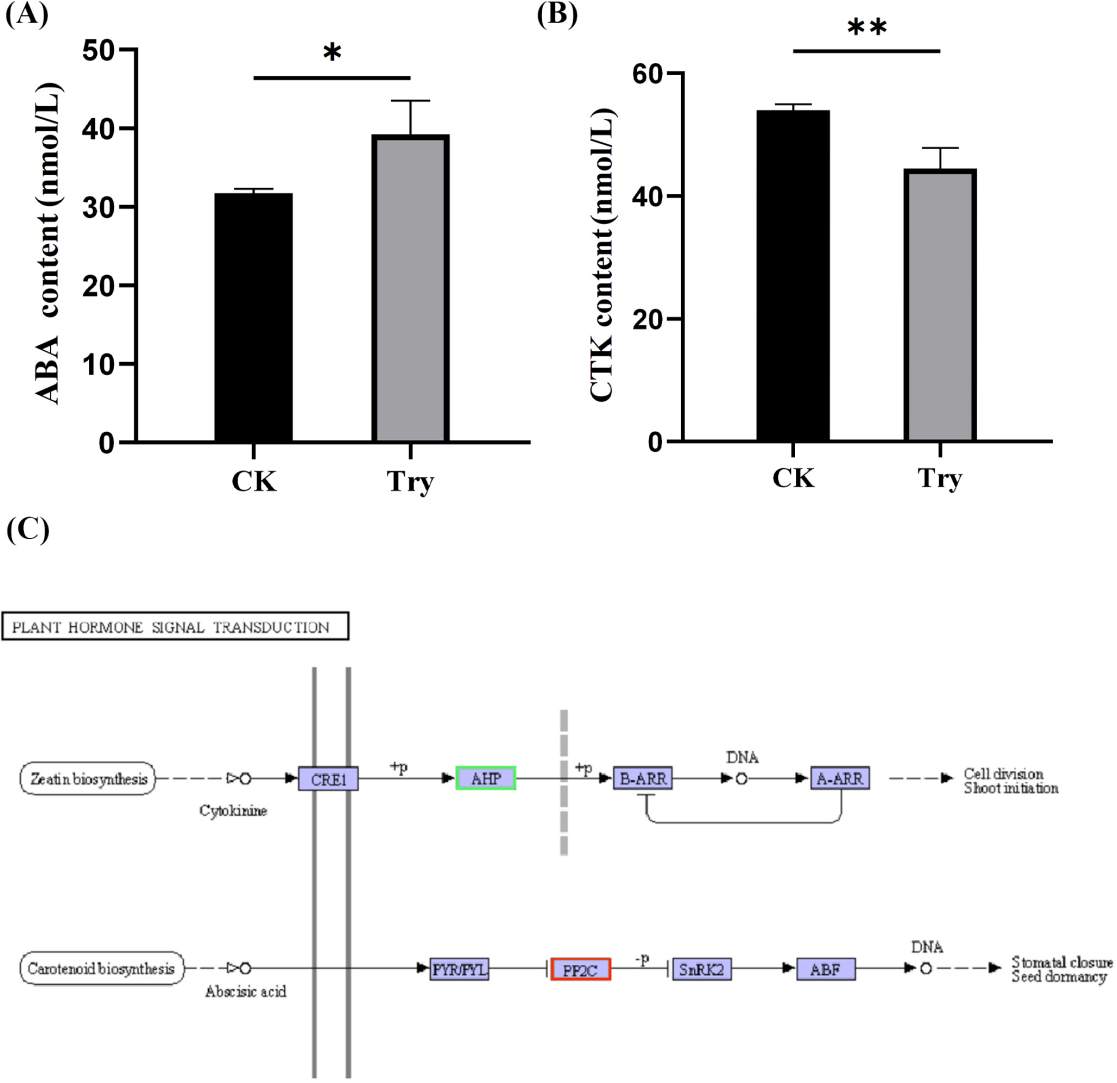
Influence of 150 μM Try on endogenous hormones of duckweed. **(A)** ABA content; **(B)** CTK content; **(C)** plant hormone signal transduction. CRE1, Cytokinin Response 1; AHP, Arabidopsis Histidine Phosphotransfer Protein; B-ARR, Type-B Arabidopsis Response Regulator; A-ARR, Type-A Arabidopsis Response Regulator; PYR/PYL, Pyrabactin Resistance/PYR1-Like; PP2C, Protein Phosphatase 2C; SnRK2, Sucrose Non-fermenting 1-Related Protein Kinase 2. (*p < 0.05, **p < 0.01).

To reveal the effect of Try on the endogenous hormone content of duckweed and the expression of its related genes, we measured the content of ABA and CTK and the expression of their related genes. Our results indicated that the ABA content significantly increased by 23.58% (*p < 0.05;* **Figure 4A**) and the CTK content significantly decreased by 17.55% (*p < 0.01;* **Figure 4B**), compared to the CK.

Transcriptome sequencing results showed that Try treatment led to increased expression of genes related to ABA production and decreased expression of genes associated with CTK production. The impact of exogenous Try on the endogenous hormone content in duckweed has been uncovered at the level of gene expression (**Figure 4C**). The above results showed that Try regulated the endogenous hormone content of duckweed by regulating gene expression, and finally regulated the growth state of duckweed.

### 3.5 Influence of 150 μM Try on ROS and POD of duckweed

**Figure 5:**
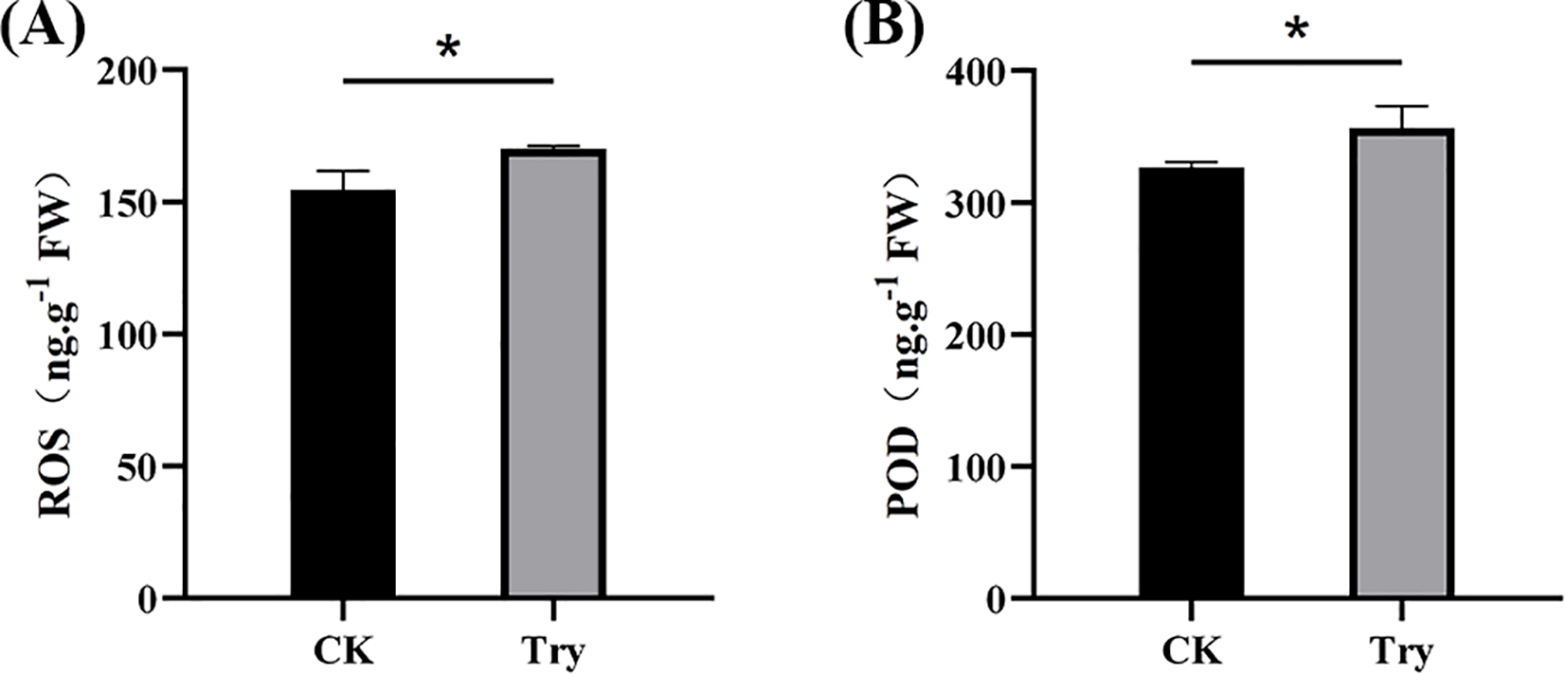
Influence of 150 μM Try on ROS and POD of duckweed. **(A)** ROS content; **(B)** POD content. (*p < 0.05).

To investigate the oxidative stress response under 150 μM Try treatment, we measured reactive oxygen species (ROS) accumulation and peroxidase (POD) activity in both experimental and CK groups (**Figure 5**). The results showed that ROS levels in the Try group were 356.444 ng/g which was 9.17% more than those in CK (326.597 ng/g). Similarly, the Try treated duckweed exhibited a POD value of 170.140 ng/g, showing a 10.11% increase compared to the CK (154.513 ng/g). These findings suggested that the Try treatment induced oxidative stress, triggering ROS overproduction and simultaneously activating the antioxidant defense system, as evidenced by enhanced POD activity to scavenge excessive ROS and mitigate cellular damage.

### 3.6 Influence of exogenous Try on Try metabolism and endogenous auxin content of duckweed

**Figure 6:**
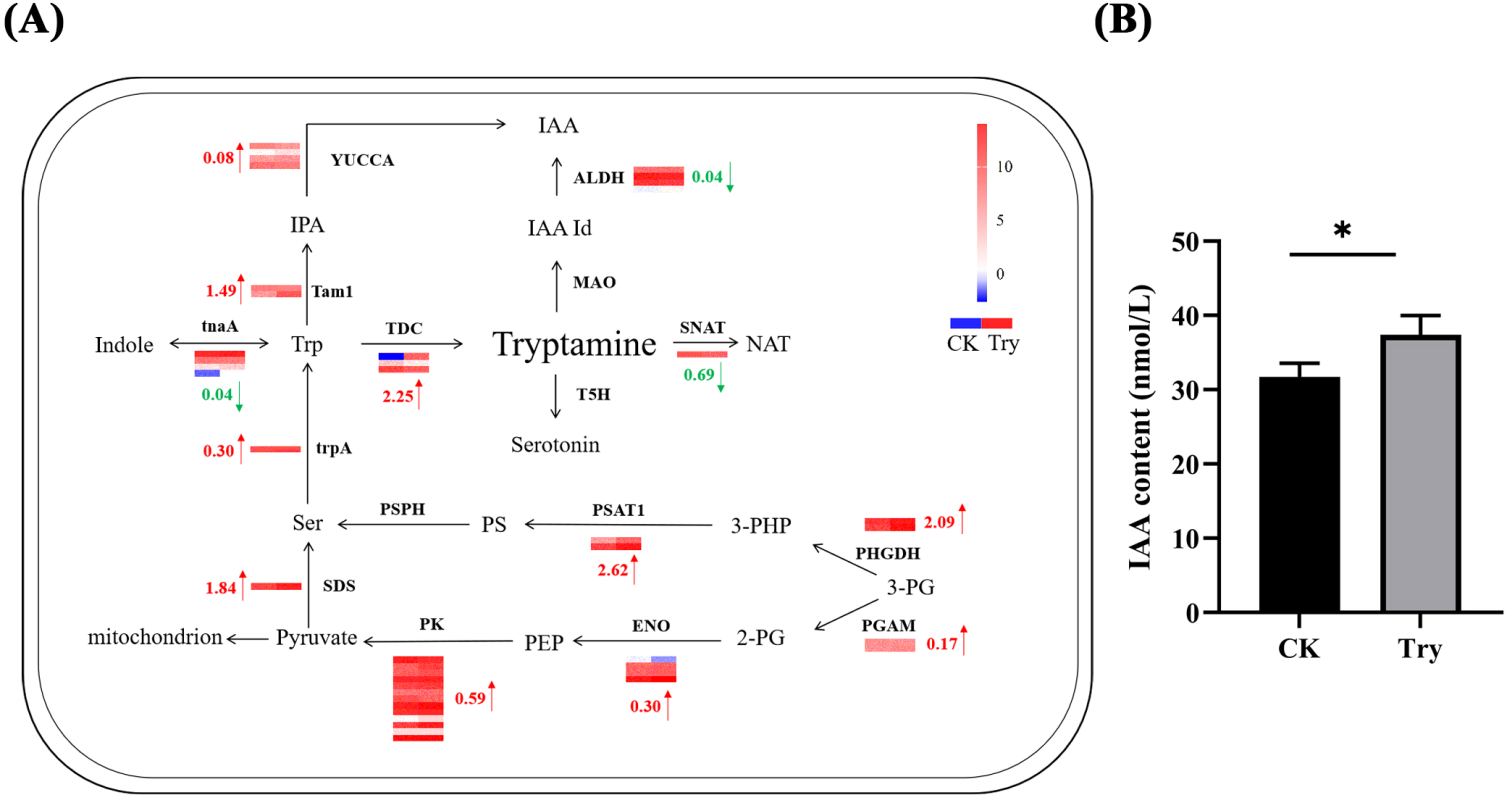
Influence of exogenous Try on Try metabolism and endogenous auxin content of duckweed **(A)** Showing the differential regulation of mRNA expression of Try pathway enzymes. Red arrows represent up-regulation, green arrows represent down-regulation. The legend colors in this figure were changed from red to blue, reflecting a decrease in log_2_ fold change values (red: high expression; blue: low expression). The left column of the heat map represents the CK group, and the right column represents the Try treatment group. The heatmap data is the log_2_ value of the readcount. PHGDH, phosphoglycerate dehydrogenase; trpA, tryptophan synthase alpha chain; PSAT1, Phosphoserine Aminotransferase 1; PSPH, phosphoserine phosphatase; TDC, tryptophan decarboxylase; tnaA, tryptophanase; SNAT, Catechol-O-MethylTransferase; TPH, tryptophan 5-monooxygenase; T5H, tryptamine 5-hydroxylase; MAO, monoamine oxidase; tam1, tryptophan aminotransferase; YUCCA, Indole-3-pyruvate monooxygenase; ALDH, aldehyde dehydrogenase; SDS, serine threonine ammonia-lyase; PGAM, 2,3-bisphosphoglycerate-dependent phosphoglycerate mutase; ENO, enolase; PK, pyruvate kinase; **(B)** The content of IAA in CK and 150 μM of exogenous Try duckweed. (*p < 0.05).

To study the effect of exogenous application of Try on the Try metabolism-related genes of duckweed, we analyzed the expression profiles of the related genes using RNA-seq data (**Figure 6A**). In the Try metabolic pathway, there were four key enzymes that directly affected Try content, they were TDC, MAO, T5H and SNAT. The expression of MAO and T5H did not change much compared with CK. However, the expression of TDC was revised up by 2.25 log_2_ Fold Change and the expression of SNAT was revised down by 0.69 log_2_ Fold Change. Combining the expression of these four enzymes, we found that exogenous Try treatment of duckweed resulted in the increase of endogenous Try content. The content of Try in senescent duckweed also increased significantly. These results indicated that external application of Try could affect the growth of duckweed. From the expression of Try metabolism-related enzymes in duckweed after external application of Try, it could be seen that the expressions of YUCCA and ALDH, two enzymes directly related to IAA generation, were changed. The expression of YUCCA was revised up by 0.08 log_2_ Fold Change and the expression of ALDH was revised down by 0.04 log_2_ Fold Change. According to the expression of these two enzymes, exogenous application of Try can increase the content of endogenous IAA in duckweed. Furthermore, endogenous IAA levels were quantified (**Figure 6B**), demonstrating a 17.76% increase in IAA content in the experimental group (37.39 nmol/L) compared to the CK group (31.75 nmol/L). Collectively, these findings suggested that aging in duckweed was linked to dynamic reprogramming of Try metabolism and auxin biosynthesis, which might influence growth regulation and stress acclimation.

## 4 Discussion

The present study provided comprehensive insights into the physiological and molecular effects of Try on *Lemna turionifera* 5511, particularly focusing on its role in growth regulation, photosynthesis and hormonal balance. Our findings revealed that Try, a polyamine derived from tryptophan, significantly influenced duckweed growth and metabolism, suggesting its potential as a regulatory molecule in plant development.

### 4.1 Try accumulation and growth regulation

Our results indicate that Try content increases in overgrown duckweed, suggesting a possible role in growth regulation. The accumulation of Try in senescent duckweed (14.76 µmol/L) compared to young duckweed (13.78 µmol/L) implies that Try may be involved in the aging process or stress responses in plants (**Figure 3B**). This is consistent with previous studies. It was shown that the levels of polyamines vary at various growth stages of plants. Try played crucial roles in plant growth, development, and stress responses (Kusano et al., 2008; Tiburcio et al., 2014). For instance, during the development processes such as flowering and cell division, there are complex interactions with plant hormones, which are crucial for the normal growth and development of plants (Kusano et al., 2008). In Arabidopsis, the SHT (spermidine hydroxycinnamoyltransferase) gene is expressed exclusively in tapetum cells of anthers (Tiburcio et al., 2014). Its product is necessary for proper pollen wall development. Under abiotic stresses such as drought and salinity, the levels of polyamines in plants increase to cope with this stress. Overexpression of ADC2 in Arabidopsis leads to putrescine accumulation, enhancing drought tolerance (Kusano et al., 2008). The downregulation of key Try-related metabolic enzymes such as PHGDH, PSPH, trpA, and SNAT in senescent duckweed further supports the idea that Try metabolism is closely linked to plant aging and growth regulation (**Figure 3A**).

The exogenous application of Try at concentrations ranging from 50 to 200 μM resulted in a dose-dependent inhibition of duckweed growth (**Figure 1A**). Notably, 150 μM Try significantly reduced the growth rate, leaf area, and chlorophyll content, indicating that Try can act as a growth inhibitor at higher concentrations (**Figures 1B–D**). This finding aligns with previous studies showing that excessive levels of certain polyamines can inhibit plant growth and development (Alcázar et al., 2010). The observed yellowing and browning of leaves in Try-treated duckweed further suggest that Try may induce oxidative stress, leading to cellular damage and growth inhibition (**Figure 1C**).

### 4.2 Try impact on photosynthesis and reactive oxygen species and peroxidase activity

The reduction in chlorophyll content and Fv/Fm in Try-treated duckweed indicates that Try negatively affects photosynthesis (**Figure 2**). The down-regulation of the genes of the proteins related to photosynthesis further supports this conclusion (**Table 1**). Although the application of polyamines can improve the photosynthesis of plants (Killiny and Nehela, 2020). Polyamines enhance the photosynthetic capacity of citrus plants by improving chlorophyll content, net photosynthetic rate and Fv/Fm. However, the high dose of PAs may destroy the structure of chloroplasts based on light conditions (Wu et al., 2012). This might be that polyamines have triggered the relevant stress response in chloroplasts, leading to excessive accumulation of ROS. When the production of ROS exceeds the clearance capacity of the chloroplast’s antioxidant system, photo-oxidative damage will be triggered.

The increase in reactive oxygen species (ROS) and peroxidase (POD) activity in Try-treated duckweed indicates that Try induces oxidative stress (**Figure 5**). ROS are known to cause cellular damage, including lipid peroxidation, protein oxidation, and DNA damage (Mittler, 2002). The increase in POD activity suggests that duckweed responds to Try-induced oxidative stress by enhancing its antioxidant defense mechanisms. Previous studies have shown that polyamines can modulate ROS levels and antioxidant enzyme activities in plants (Groppa et al., 2003). These observations indicates that Try triggers the stress response of plants, leading to a decline in photosynthesis and a reduction in chlorophyll content in duckweed. Duckweed eliminates the negative impact of ROS by increasing the content of POD.

### 4.3 Hormonal regulation and stress responses

Our study also highlights the role of Try in modulating endogenous hormone levels, particularly abscisic acid (ABA) and cytokinin (CTK) (**Figures 4A, B**). The significant increase in ABA content and decrease in CTK content in Try-treated duckweed suggests that Try may induce stress responses in plants. ABA is known to play a crucial role in plant stress responses, including stomatal closure and seed dormancy (Finkelstein, 2013), while CTK is involved in delaying senescence and maintaining chlorophyll content (Zwack and Rashotte, 2015). It has also been proposed that polyamines are involved in plant tolerance and acclimation to drought by modulating ABA and CTK biosynthesis (Asija et al., 2023; Napieraj et al., 2023). Under drought conditions, polyamines increase the ABA content in plants by inducing the expression of key genes for ABA biosynthesis (such as NCED), promote stomatal closure to reduce water loss, and enhance the plants’ response to drought stress by regulating the expression of related genes in the ABA signal transduction pathway. Meanwhile, polyamines can also cause changes in CTK content, regulate physiological processes such as cell division, chlorophyll biosynthesis and nutrient absorption, and control the growth and photosynthetic efficiency of plants (Napieraj et al., 2023). In our studies, ABA-related genes were upregulated and CTK-related genes were down-regulated in duckweed treated with Try, which supported the view that Try induced a stress response, triggers changes in endogenous related hormones in duckweed, and leads to growth inhibition and aging (**Figure 4C**).

## 5 Conclusion

In conclusion, our study demonstrates that Try plays a complex role in regulating duckweed growth, photosynthesis, and stress responses. The accumulation of Try in older duckweed, its growth-inhibiting effects at higher concentrations, and its impact on hormonal balance and oxidative stress responses highlight its potential as a regulatory molecule in plant development. Further research is needed to explore the molecular mechanisms underlying the effect of Try and its potential applications in agriculture and environmental management.

## Data Availability

The datasets presented in this study can be found in online repositories. The names of the repository/repositories and accession number(s) can be found in the article/supplementary material.
