# Correction: Effects of tryptamine on duckweed growth

**DOI:** 10.3389/fpls.2025.1663336

**Published:** 2025-08-01

**Authors:** Qiqi Di, Wenqian Han, Yujie Han, Sizheng Liu, Yi Hu, Ziyang Qu, Yumeng Jiang, Weibo Sun, Ting Qiu, Lin Yang

**Affiliations:** ^1^ Tianjin Key Laboratory of Animal and Plant Resistance, College of Life Sciences, Tianjin Normal University, Tianjin, China; ^2^ Tsinghua-Peking Center for Life Sciences, College of Life Sciences, Tsinghua University, Beijing, China

**Keywords:** tryptamine, *Lemna turionifera* 5511, growth regulation, photosynthesis, oxidative stress, hormonal balance

In the published article, there was an error in the **Funding** statement. The funding number for the undergraduate innovation training program was incorrectly stated in the original text “undergraduate innovation training program (202210065168).” The correct **Funding** statement appears below.

“The author(s) declare that financial support was received for the research and/or publication of this article. Present research has been supported by National Natural Science Foundation of China (No.32471699, No. 32071620), Tianjin Natural Science Foundation of Tianjin (23JCYBJC00540), Tianjin Education Reform Project (B231006511) and undergraduate innovation training program (202410065040).”

The authors apologize for this error and state that this does not change the scientific conclusions of the article in any way. The original article has been updated.

